# Medium term health and quality of life outcomes in a cohort of children with MIS-C in Cape Town, South Africa

**DOI:** 10.3389/fped.2024.1465976

**Published:** 2025-01-28

**Authors:** Frank Phoya, Claire Butters, Timothy F. Spracklen, Hanna L. Kassa, Hamza van der Ross, Chris Scott, Kate Webb

**Affiliations:** ^1^ Division of Rheumatology, Department of Paediatrics and Child Health, University of Cape Town, Cape Town, South Africa; ^2^ Cape Heart Institute & Children’s Heart Disease Research Unit, University of Cape Town, Cape Town, South Africa; ^3^ Crick African Network, Francis Crick Institute, London, United Kingdom

**Keywords:** MIS-C (multisystem inflammatory syndrome in children), quality of life, juvenile idiopathic arthiritis, South Africa, physical deficits

## Abstract

**Background:**

Multisystem inflammatory syndrome in children (MIS-C) is a disease that occurs after exposure to severe acute respiratory syndrome coronavirus 2 (SARS-CoV-2). Its short-term effects have been documented but little data exist on the longer term effects of MIS-C on the health and quality of life (QOL) of patients. The objective of this study was to assess the long-term effects of MIS-C on the QOL of children.

**Methods:**

This study was a descriptive prospective study. We included 24 participants with previous MIS-C and 20 children with juvenile idiopathic arthritis (JIA) as a positive comparator group. All children were examined and completed a paediatric quality of life (PedsQL) generic inventory score. This score was used to evaluate the School Functioning, Social, Emotional, and Physical QOL domains.

**Results:**

All participants with previous MIS-C made a full recovery, with no medical complaints, and normal physical examinations after a median of 705 days post acute diagnosis. The PedsQL inventory revealed that 16.7% of the children with previous MIS-C showed a deficit in the physical domain compared to 60% of the children with JIA (*p* < 0.001). 12.5% of the children with previous MIS-C had a deficit in their psychosocial domain which included emotional, social, and educational scores, compared to 40% of the children with JIA (*p* = 0.035).

**Conclusions:**

In a cohort of 24 South African children with previous MIS-C, no medical complications were reported. A small proportion felt a prolonged effect on their QOL even after making a full recovery, although this was not as severe as children with JIA, a known chronic disease that affects QOL. This highlights the need to continue to follow up these patients and offer more comprehensive long-term care.

## Background

In 2020, a hyperinflammatory syndrome with multiorgan involvement, potentially linked to Coronavirus disease (COVID-19) emerged among clusters of children, resembling Kawasaki disease, but with notable clinical and diagnostic distinctions (1). While data on this syndrome, Multisystem Inflammatory Syndrome in Children (MIS-C), remains rare in Africa, a cohort study done in Cape Town, South Africa helped to estimate its incidence and characteristics, estimating an incidence of 22 cases per 100,000 SARS-CoV-2 exposures (2–4). MIS-C presents with fever, inflammation, and involvement of multiple organ systems, often including gastrointestinal symptoms, with lower mortality rates compared to classic Kawasaki disease (4, 5).

Despite the relatively low mortality rates, concerns over long-term impacts on health and quality of life (QOL) post-MIS-C have emerged (6, 7). Very few studies have investigated long-term health and QOL in children after MIS-C. In the UK, Penner et al. reported that almost 20% of children with previous MIS-C had deficits in the psychosocial QOL with persistent emotional and physical impairment in some children (8). There are no studies that compare the QOL of children with previous MIS-C to an unwell comparator group from the same setting, in order to contextualise the residual impairment. Research on the long-term effects of MIS-C, particularly on QOL, remains limited, especially in Africa.

Here, we aimed to describe the long-term effects of MIS-C on the health and QOL of a cohort of children in Cape Town, South Africa. We included children with juvenile idiopathic arthritis (JIA), a chronic disease with a known severe effect on QOL (9) as a positive comparator group in order to contextualise these findings.

## Methods

We performed a descriptive prospective longitudinal cohort study in which participants diagnosed with MIS-C between March 2020 and January 2022 were recalled and offered participation between January 2023 and December 2023. Children with JIA were recruited as a convenience comparator group during the same period. The study was conducted at Red Cross War Memorial Children's Hospital, which is a government-funded, tertiary-level referral hospital located in Cape Town, South Africa. This study was approved by the University of Cape Town Human Research Ethics Committee (HREC 112/2012; 599/2020).

Inclusion criteria included:

• Children and young people (CYP) aged less than 18 years.
• Previous diagnosis of MIS-C between March 2020 and January 2022 (Diagnosed according to the WHO, MIS-C criteria) (Supplementary Table 1)
• 6 months or more post-disease onset (MIS-C)
• CYP attending paediatric rheumatology clinics with a diagnosis of JIA according to the treating physician

The exclusion criteria were:

• Alternative diagnoses (e.g., Systemic lupus erythematous)
• Chronic, co-morbid diagnosis that may affect the QOL (e.g., HIV, malignancy)

CYP (or their parents where appropriate) who had agreed to be contacted again by researchers during their initial consent for a paediatric biorepository study and met the inclusion and exclusion criteria were contacted telephonically to return for a repeat clinical follow-up visit.

During the visit, variables were collected, including demographics (i.e., sex, age) and current complaints (i.e., joint pain, recent infection). Clinical data was collected [weight, height, and vital signs (temperature, respiratory rate, heart rate and blood pressure)] and recorded in a case report form (CRF) (Supplementary Appendix B2). A brief history was taken and a follow-up on their health since the last clinical visit was performed. A general examination was done reviewing the neurological, respiratory, cardiovascular, gastroenterology, and musculoskeletal system and recorded in the CRF (Supplementary Appendix B2). At the end of the visit, patients or guardians were asked to fill in a paediatric QOL Inventory/questionnaire (PedsQL; Supplementary Appendix B1). “PedsQL is a 23-item generic health status instrument that is used to assess five domains of health (physical functioning, emotional functioning, psychosocial functioning, social functioning, and school functioning)” (10). “The tool can be used as a child self-report or parent proxy-report format. Child self-report includes ages 5–7, 8–12, and 13–18 years. Parent proxy report includes ages 2–4 (toddler), 5–7 (young child), 8–12 (child), and 13–18 (adolescent), and assesses parents’ perceptions of their child's health-related quality of life (HRQOL)”. “A 5-point response scale is utilized across child self-report for ages 8–18 and parent proxy-report (0 = never a problem; 1 = almost never a problem; 2 = sometimes a problem; 3 = often a problem; 4 = almost always a problem)”. “To further increase the ease of use for the young child self-report (ages 5–7), the response scale is reworded and simplified to a 3-point scale (0 = not at all a problem; 2 = sometimes a problem; 4 = a lot of a problem)”. “Items were reverse scored and linearly transformed to a 0–100 scale (0 = 100, 1 = 75, 2 = 50, 3 = 25, 4 = 0) so that higher scores indicate better HRQOL”. “Scale scores were computed as the sum of the items divided by the number of items answered (to account for missing data). If more than 50% of the items in the scale are missing, the scale score was not computed”. “A score of less than 80% was indicative of a deficit in that particular domain”. The Psychosocial Score (15 items), the mean was computed as the sum of the items divided by the number of items answered in the School Functioning, Social, and Emotional Subscales (10).

In children less the 12 years old, the questionnaire was completed by their guardian/parent. We asked children older than 12 years old to fill out the questionnaire independently.

The statistical analyses were performed using SPSS (version 28.0.1.1). Simple descriptive statistics were used to summarize variables. Data was coded appropriately e.g., scores as ordinal data (e.g., PedsQL) or scale numerical data (weight, height, etc.) as applicable before entering into the analytical program. As per PedsQL guidelines, ordinal scores were transformed into numerical values (11). Numerical data such as age were presented as mean (Standard Deviation) or median (Inter-Quartile Range) depending on data normality. Categorical variables were presented as proportions or percentages. Data between groups (MIS-C and JIA) were compared by non-parametric comparator testing as appropriate, such as Mann-Whitney U tests or Fisher's exact tests.

### Disease definitions

MIS-C (Multisystem inflammatory syndrome in children).

## Results

Of the 64 MIS-C cases diagnosed at Red Cross War Memorial Children's Hospital between 2020 and 2022, 24 children with previous MIS-C and 20 children with JIA agreed to take part in the study.

The clinical characteristics of the larger cohort have been described (4). Of the 24 MIS-C participants who were followed up in this study, 66.7% were male. At the time of diagnosis of MIS-C, the median age was 8.2 years (min = 1.4; max = 14.2) and fever, rash, conjunctivitis, tachycardia, hypotension, abdominal pain, diarrhoea, headache and arthritis were common features (Table 1). Summary clinical laboratory data are supplied in Table 1. Central nervous system (CNS) and renal disease were present in 25% and 16.7% of the patients respectively. Coronary artery dilatation was recorded in one patient, with a median ejection fraction of 61% (min = 32; max = 70) in the cohort. During admission, 87.5% of the children received antibiotics, 70.8% received intravenous immunoglobulin (IVIG) at 2 g/kg and 58.3% received IV methylprednisolone (30 mg/kg per day for 3 days). One third (29.2%) required ICU admission or inotropic blood pressure support (33.3%). All patients made a full recovery and were discharged home, with no deaths recorded. The average in-hospital stay was 8 days (SD- 3.7, min = 5; max = 21) (Table 1).

**Table 1:** MIS-C and JIA demographics and clinical features.

MIS-C	
Demographics at diagnosis	*n* = 24
Gender	66.7% (16)
Age (median)	8.2 year (min = 1.4; max = 14.2)
Median time since diagnosis	705 days (min-255; max = 1,004)
Clinical features at diagnosis	
Fever	100% (24)
Rash	100% (24)
Abdominal pain	100% (24)
Tachycardia	91.7% (22)
Conjunctivitis	79.2% (19)
Hypotension	54.2% (13)
Headache	45.8% (11)
Diarrhoea	41.7% (10)
Arthritis	37.5% (9)
CNS disease	25% (6)
Lung disease	20.8% (5)
Renal disease	16.7% (4)
Bleeding	4.2% (1)
Coronary artery aneurysm present	4.2% (1)
Median ejection fraction	61% (min = 32; max = 70)
Median CRP	173 mg/dl (min = 103; max = 511)
Median white cell count	17.2 × 10^9^/L (min = 8.6; max = 59.9)
Median Pro BNP	1,319 pg/ml (min = 24; max = 48,172)
Initial management and outcomes	
1st line antibiotics	87.5% (21)
IVIG 2 g/kg first dose	70.8% (17)
IVIG 1 g/k first dose	16.7% (4)
IVIG 2 g/kg second dose	4.2% (1)
Methylprednisone	58.3% (14)
ICU admission	29.2% (7)
Inotropes	33.3% (8)
Discharged	100% (24)
Median duration of admission	7 days (min = 5; max = 21)
JIA	
Demographics at recruitment	*n* = 20
Gender (male)	45% (9)
Age (median)	14.3 year (min = 3.1; max = 17.1)
Duration since diagnosis	1.6 years (min = 0.4; max = 10)
Median time since diagnosis	591 day (min = 27; max = 3,693)
Type of JIA	
ERA	30% (6)
Oligo	20% (4)
Poly	20% (4)
PsJIA	20% (4)
Sys	10% (2)

CRP, C reactive protein; Pro BNP, Pro brain natriuretic peptide; MIS-C, multisystem inflammatory syndrome in children; JIA, juvenile idiopathic arthritis; IVIG, intravenous immunoglobulin; IUC, intensive care unit; ERA, enthesitis related arthritis; Oligo, oligoarticular arthritis; Poly, polyarticular arthritis; PsJIA, psoriatic associated juvenile idiopathic arthritis; Sys, systemic arthritis.

The median time to the study visit from the acute diagnosis of MIS-C was 705 days (min 255; max 1,004). The minimum age at the time of the study visit was 3 years and the maximum was 16.5 years. All children were well at study visit, with no admissions or illnesses of note since their admission with MIS-C, normal growth, normal vital signs and a normal clinical examination.

Out of the 20 recruited JIA participants in the comparator group, 55% were female. The median age was 14.3 years (min = 3.1; max = 17.3). The median time since the diagnosis of JIA was made was 591 days (min = 27; max = 3,693). The most common type of JIA was enthesitis related arthritis (ERA), at 30% (Table 1). No other significant clinical problems apart from pain were reported at the time of recruitment.

### Quality of life assessment MIS-C and JIA

#### Physical domain

**Figure 1:**
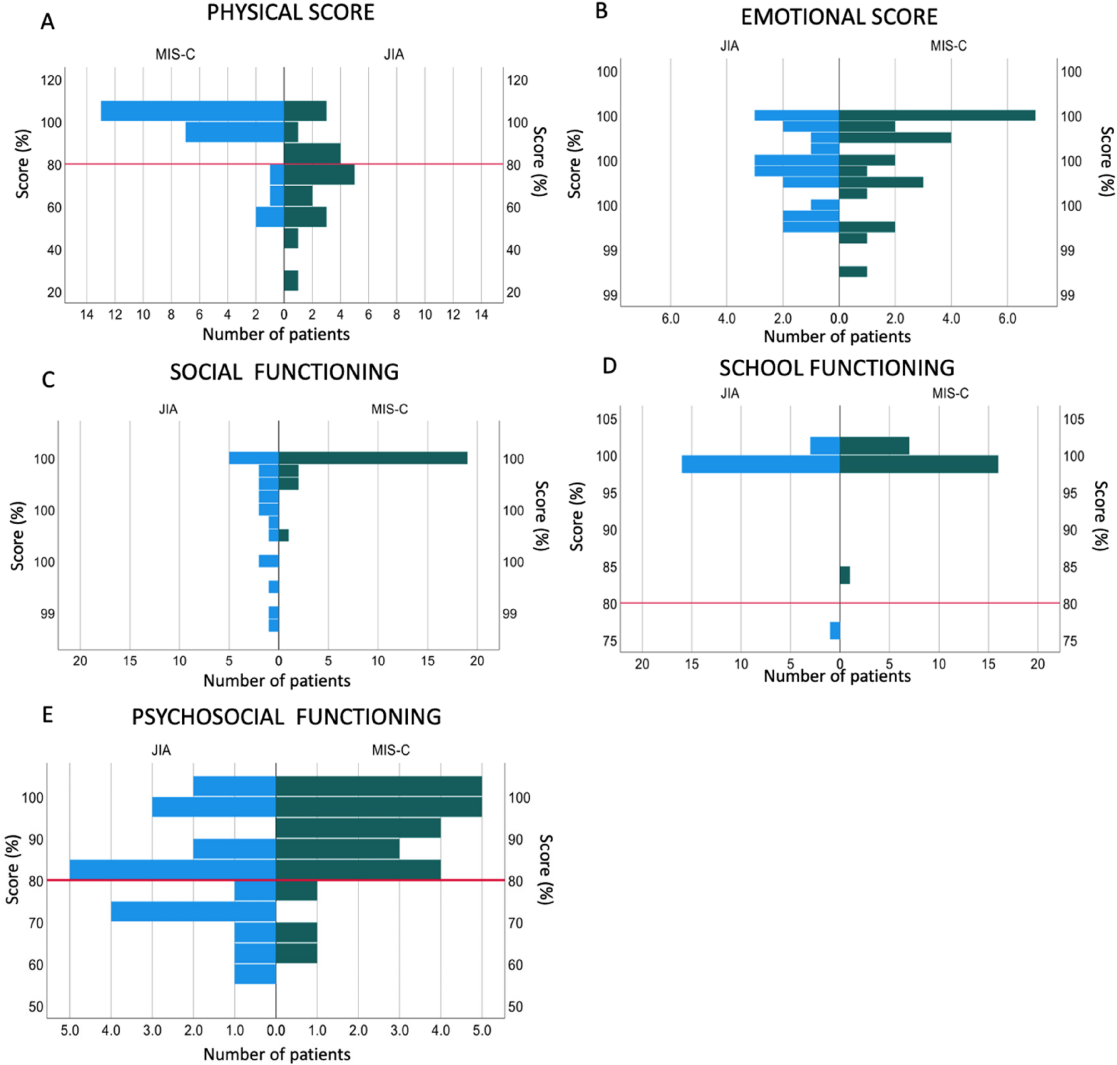
Quality of life assessments in children with previous MIS-C compared to children with JIA. **(A)** Physical domain score, **(B)** Emotional domain score, **(C)** Social functioning domain score, **(D)** School functioning domain score, **(E)** Psychosocial functioning domain score. The red line indicates 80%, scores below which indicate a deficit in that domain.

Four out of 24 (16.6%) participants with MIS-C showed an overall deficit (score of less than 80%) in the physical domain as compared to 12/20 (60%) of participants with JIA (*p* = 0.001) (Figure 1A). These included problems with walking (“often”-3/24; “almost always”- 1/24), running (“often”-1/24; “almost always”- 1/24). Compared to participants with MIS-C, participants with JIA reported more difficulty participating in active play (*p* = 0.001), doing chores (*p* = <0.001) and reported feeling tired (*p* = 0.001) (Supplementary Table 2).

#### Emotional domain

A summary percentage score for the emotional domain showed that no participant with previous MIS-C or JIA scored below 80%, showing no deficit in that field (Figure 1B) with no difference between the two groups (Supplementary Table 3).

Participants with JIA more frequently reported feelings of anger due to their condition than participants with MIS-C (*p* = 0.022) and were also found to be more worried about what would happen to them due to their disease than participants with previous MIS-C (*p* = 0.004) (Supplementary Table 3).

#### Social domain

In the social domain, participants with JIA showed more difficulties in all the 4 reported areas than participants with previous MIS-C. Participants with JIA had more difficulty getting along with other children (*p* = 0.003), doing things that their peers can do (*p* = <0.001), and keeping up when playing with their peers (*p* = <0.001) (Supplementary document Figure 4).

The summary percentage score revealed no participant scored below 80% in both groups; however, there was still a significant difference between the two groups (*p* = <0.001) (Supplementary Table 4).

#### School domain

No participants with MIS-C showed a deficit in the school domain and only 1/20 (5%) of participants with JIA showed a deficit, with no overall difference (*p* = 0.31) (Figure 1D; Supplementary Table 5). Participants with JIA more frequently reported missing school to go to the doctor (*p* = 0.003) (Supplementary Table 5; Supplementary Figure 5).

#### Psychosocial domain

The psychosocial domain was made up of a combined score of the emotional, social and school domain summary percentage scores.

Twelve percent (3/24) of participants with MIS-C had a deficit in the psychosocial domain compared to 40% of children with JIA (*p* = 0.035) (Figure 1E; Supplementary Table 6).

## Discussion

In this study, we recruited 24 participants with previous MIS-C and investigated their health and QOL after a median of 2 years from diagnosis. We compared these children to a group of children from the same setting with JIA, a known chronic and painful disease with expected deficits in multiple QOL domains (9).

All of the participants who had MIS-C previously made a full recovery and had no significant medical complications, which matches the good long-term outcomes in MIS-C reported elsewhere (6, 7).

Participants with previous MIS-C did however present with deficits in multiple QOL domains, although these were universally less severe than the deficits seen in children with JIA. Sixteen percent of participants with previous MIS-C in this study had difficulty in the physical domain and 12% showed difficulty in the psychosocial domain. This was similar to the study done by Penner et al. (8) in the UK, who showed that 13% and 18% of participants had a deficit in the physical and psychosocial domain respectively. Surprisingly, the cohort in the current study showed no significant deficit in the emotional domain as was reported in the UK study (8), which may reflect an emotional resilience in this cohort or could be due to the different time period between acute disease and study visit in the two studies.

Sixteen percent of participants with MIS-C had a deficit in the physical domain, which was lower than in participants with JIA (60%) (12). Participants with JIA reported more anger and worry than participants with previous MIS-C, which may be expected due to the chronicity of the disease.

There were no differences in scores in the social and school domain. Participants with JIA were more likely to find it difficult to get along with other children, the also found it difficult doing things that their peers were doing and difficult to keep up when playing with their peers, as expected (12).

A small sample size was one of the major limitations of this study. There are however very few long-term outcome data reported in MIS-C, and even fewer from Africa. Another limitation is the comparator group chosen; this study used only a “positive” control group, as it is known that children with JIA have low overall health-related QOL, in all aspects of physical health, psychosocial health, emotional functioning, and school functioning (9). Future research is needed to also compare the long-term QOL in children with MIS-C to a cohort that has recovered from a similar acute, monophasic disease and healthy children from the same setting. As the COVID-19 pandemic abates, we believe it is vital to continue to monitor children with MIS-C in the long term.

## Conclusions

In a cohort of 24 African children with previous MIS-C, no medical complications were reported. A small proportion felt a prolonged effect on their QOL even after making a full recovery, which was less severe than in children with JIA. This highlights the need to continue to follow up these patients and offer more comprehensive long-term care.

## Data Availability

The raw data supporting the conclusions of this article will be made available by the authors, without undue reservation.
