# Supplementary material for: Medium term health and quality of life outcomes in a cohort of children with MIS-C in Cape Town, South Africa
Source: Front Pediatr. 2025 Jan 28;12:1465976. doi: 10.3389/fped.2024.1465976 (PMC11843660; doi:10.3389/fped.2024.1465976)
Supplement: Supplementary file 1 [file Presentation1.pptx]

## Slide 1
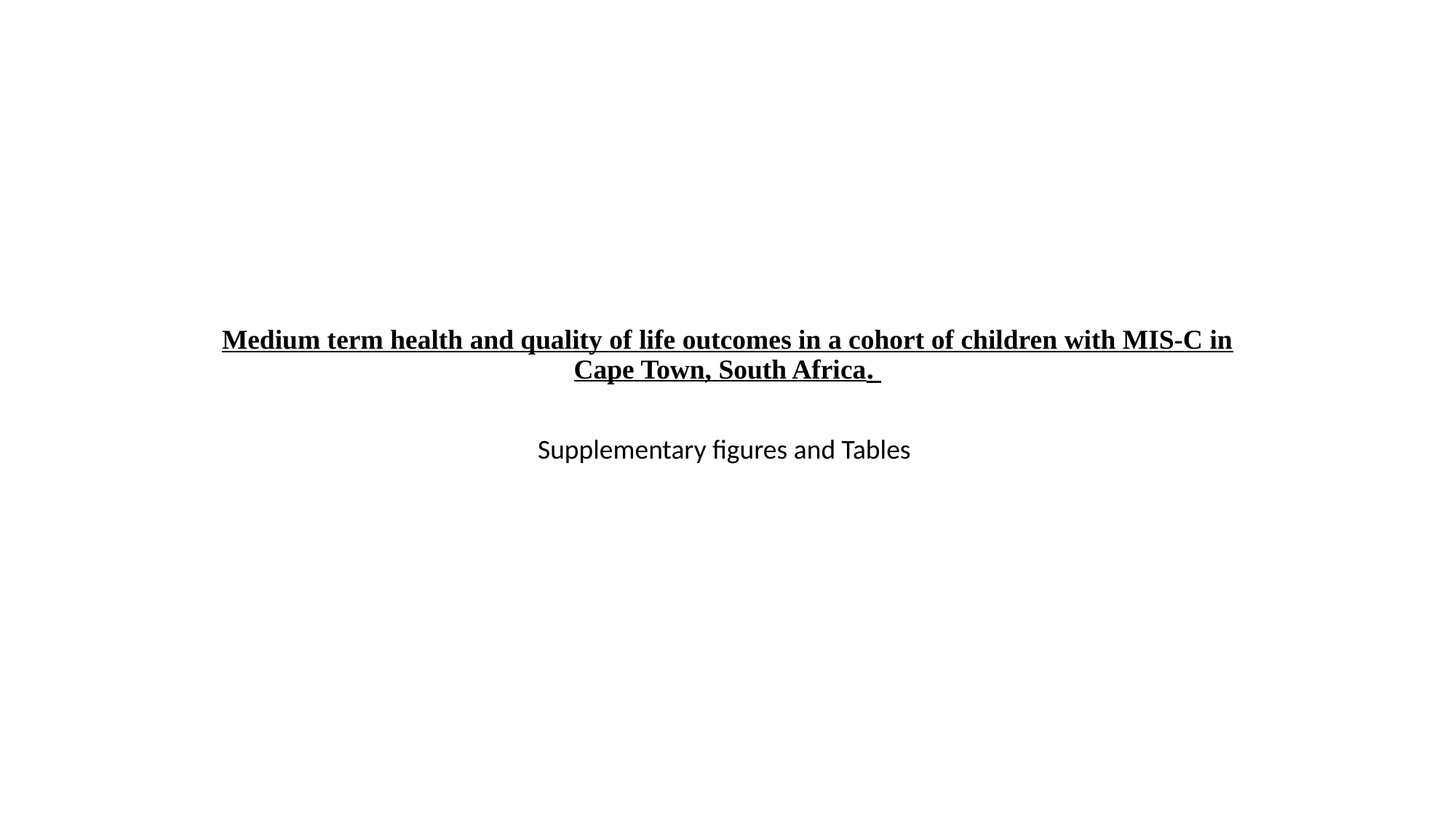

# Medium term health and quality of life outcomes in a cohort of children with MIS-C in Cape Town, South Africa.
Supplementary figures and Tables

## Slide 2
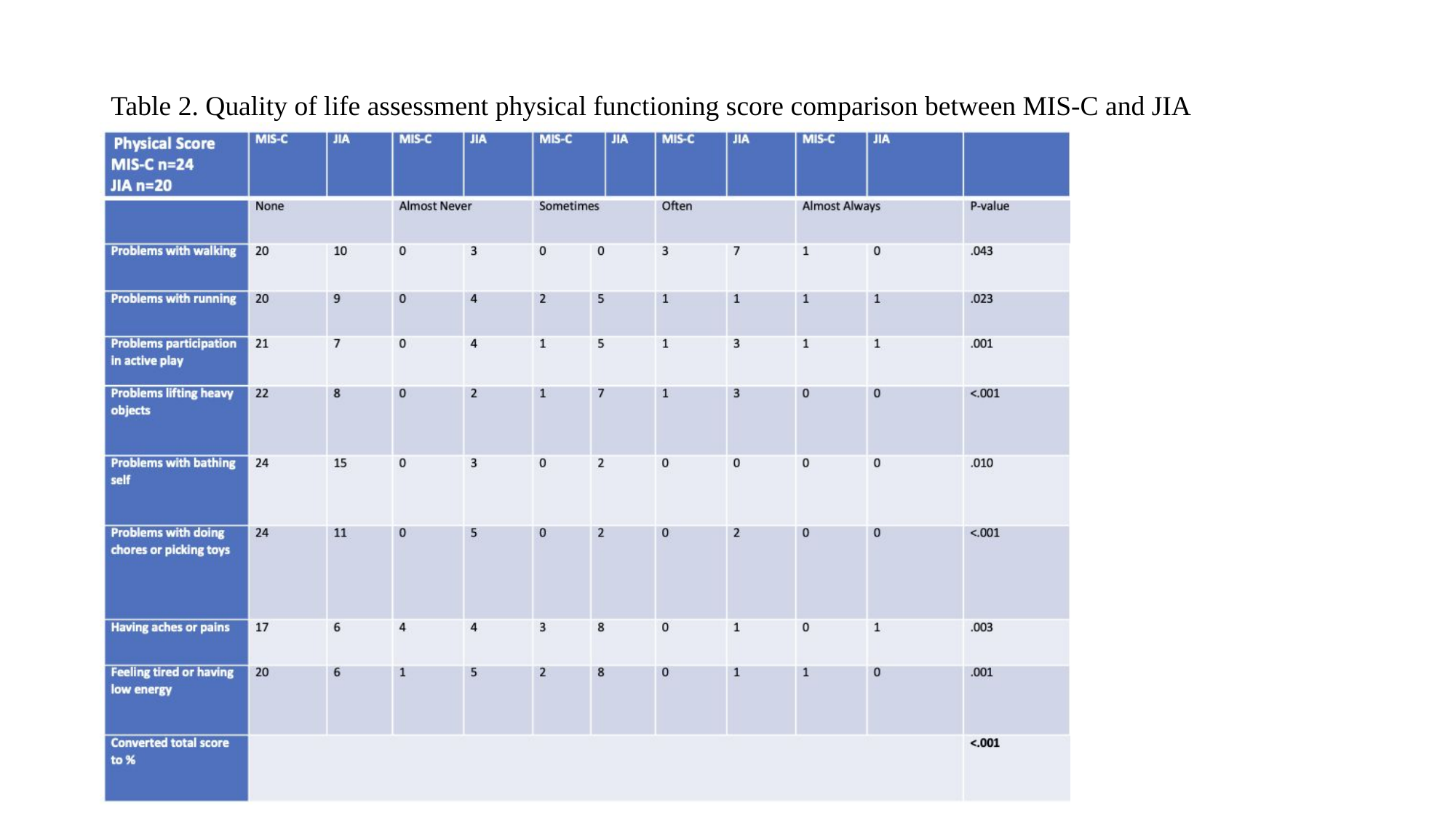

# Table 2. Quality of life assessment physical functioning score comparison between MIS-C and JIA

## Slide 3
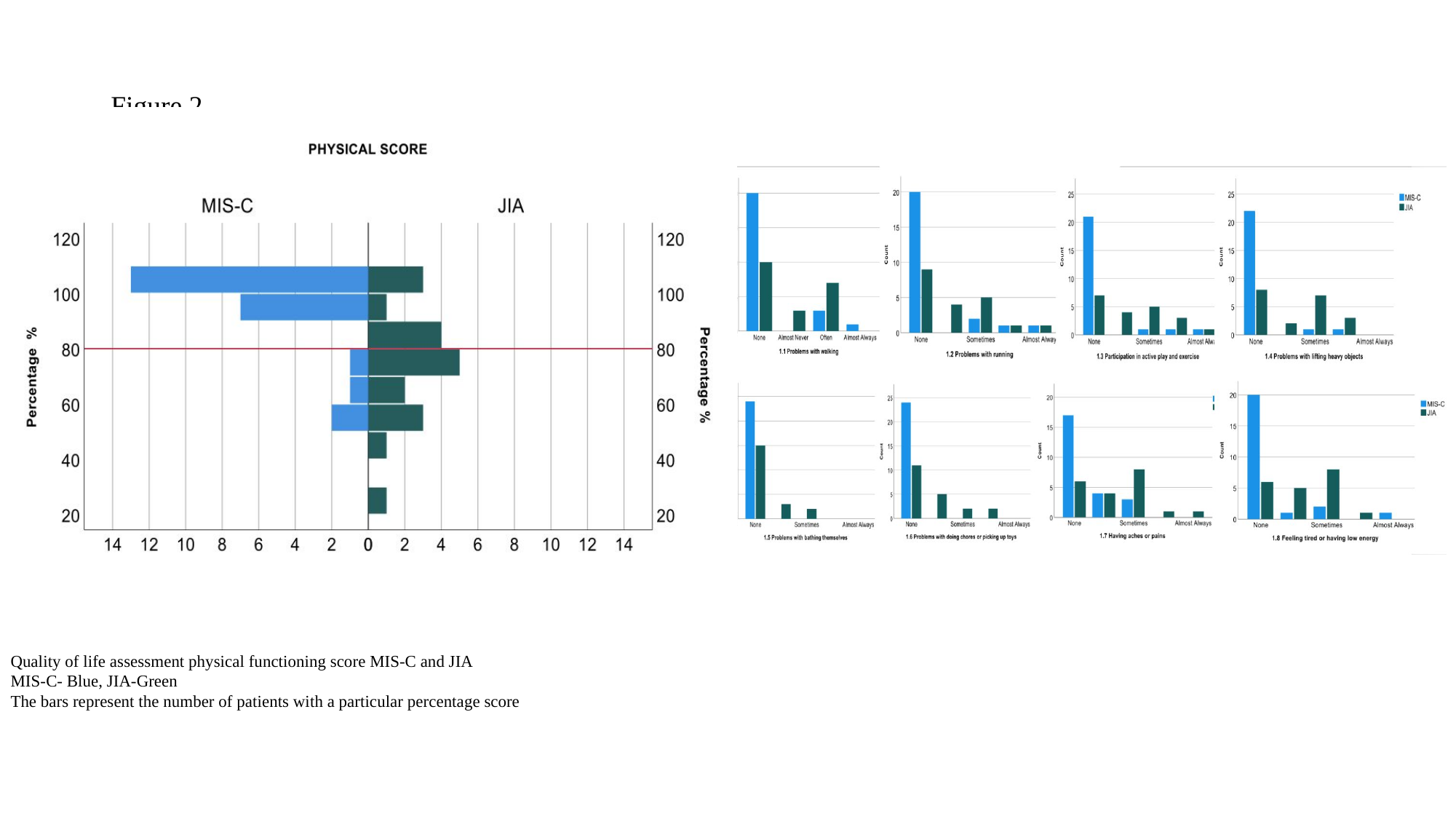

# Figure 2.
Quality of life assessment physical functioning score MIS-C and JIA
MIS-C- Blue, JIA-Green
The bars represent the number of patients with a particular percentage score

## Slide 4
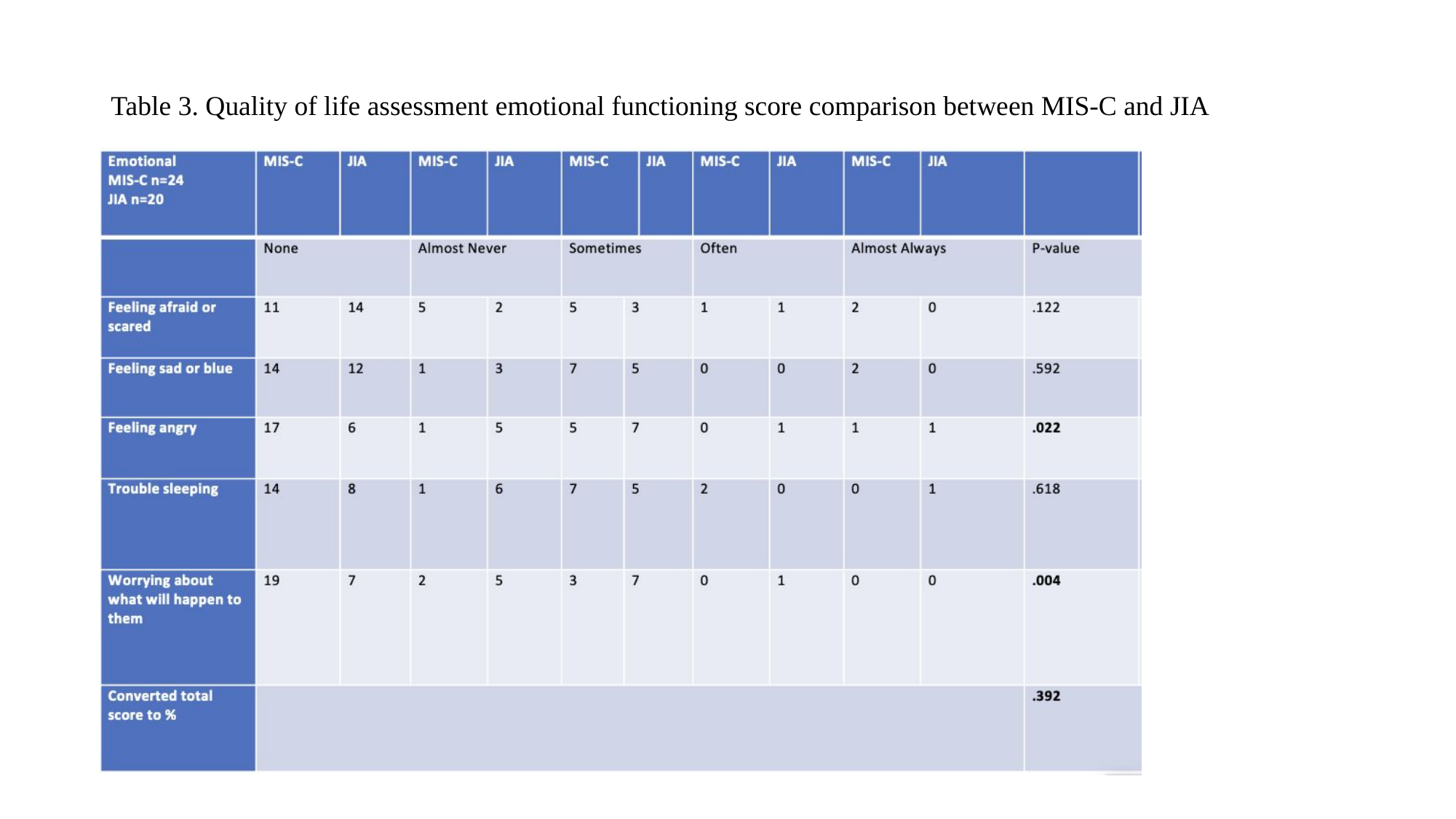

# Table 3. Quality of life assessment emotional functioning score comparison between MIS-C and JIA

## Slide 5
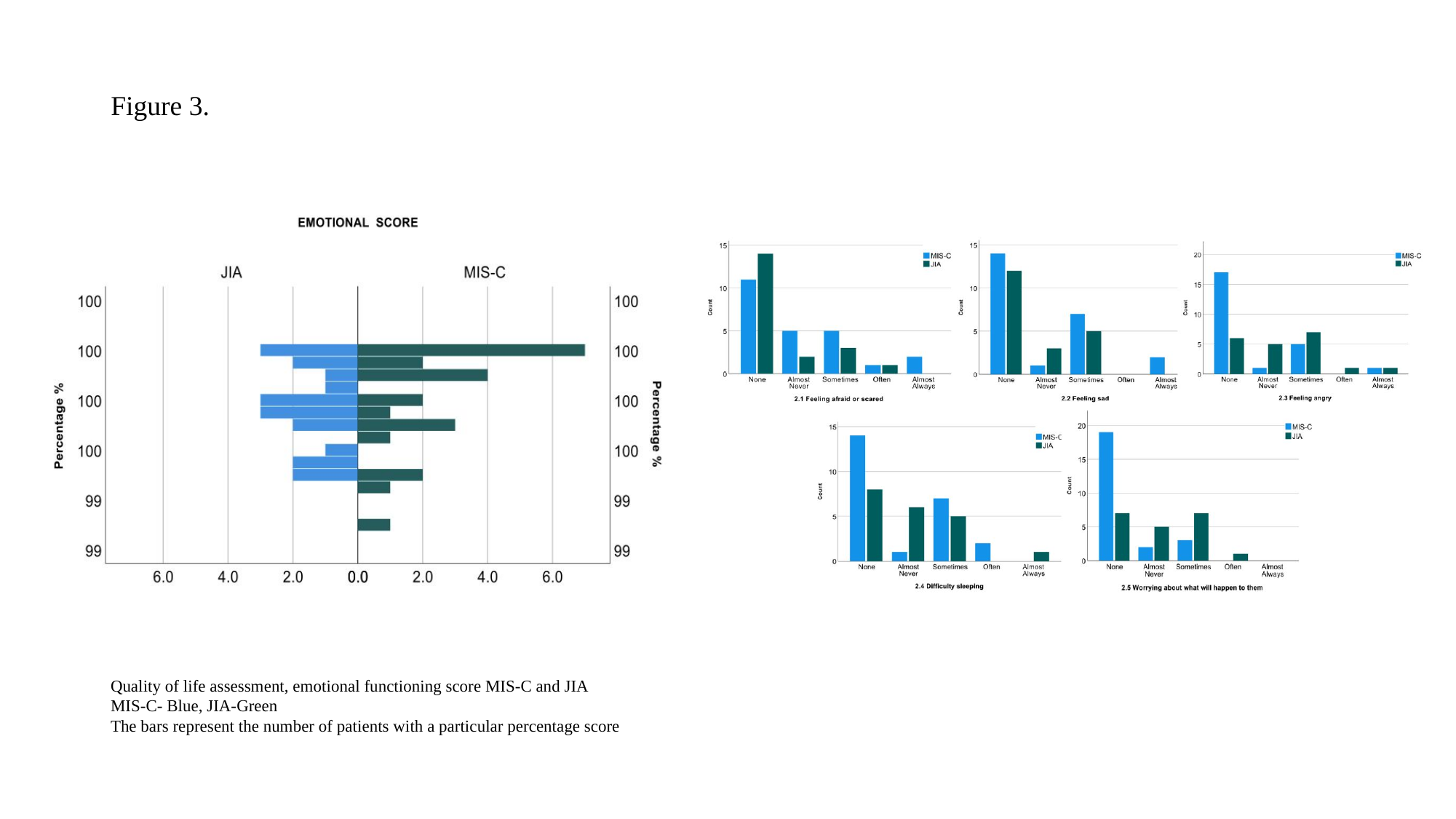

# Figure 3.
Quality of life assessment, emotional functioning score MIS-C and JIA
MIS-C- Blue, JIA-Green
The bars represent the number of patients with a particular percentage score

## Slide 6
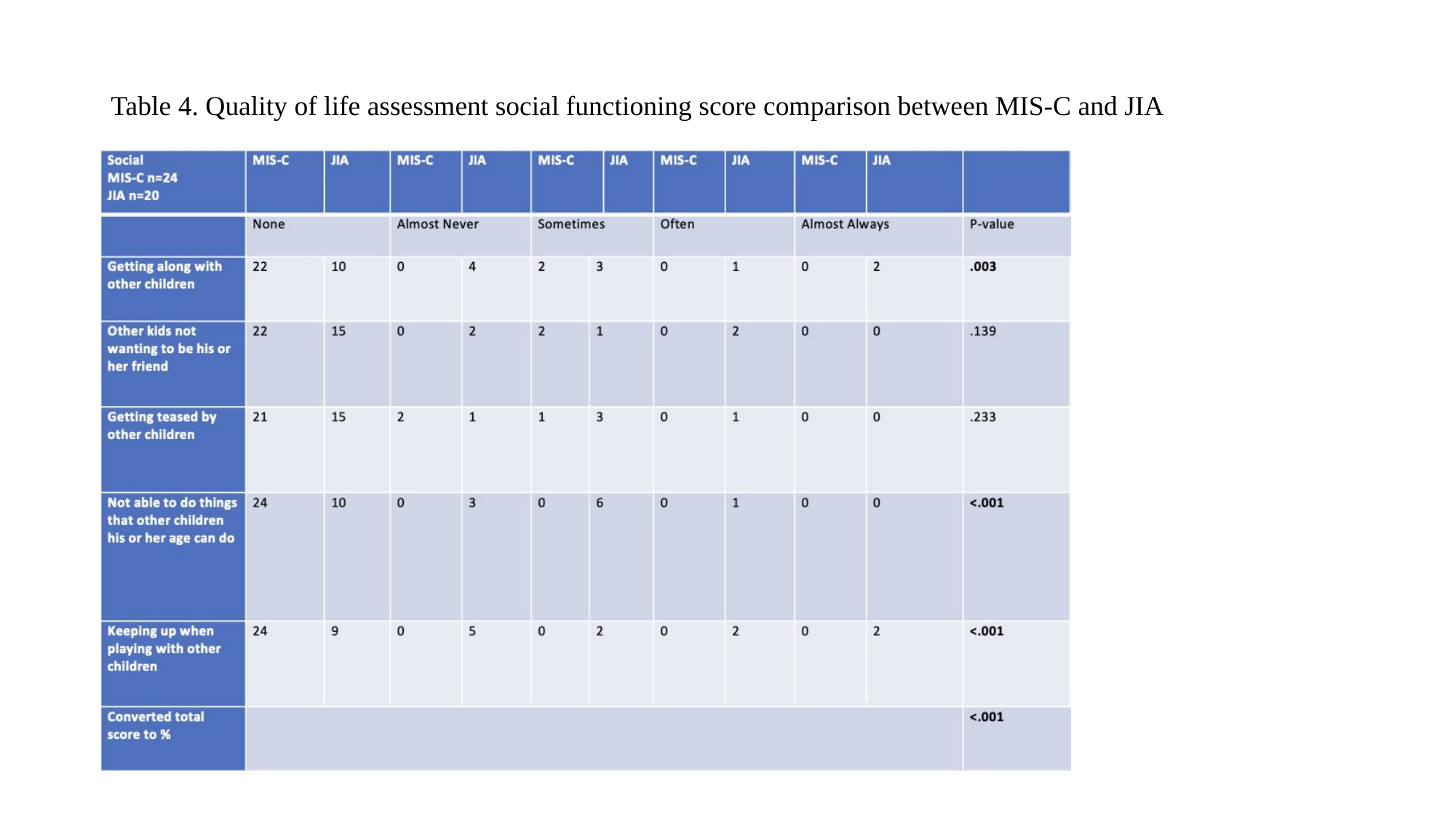

# Table 4. Quality of life assessment social functioning score comparison between MIS-C and JIA

## Slide 7
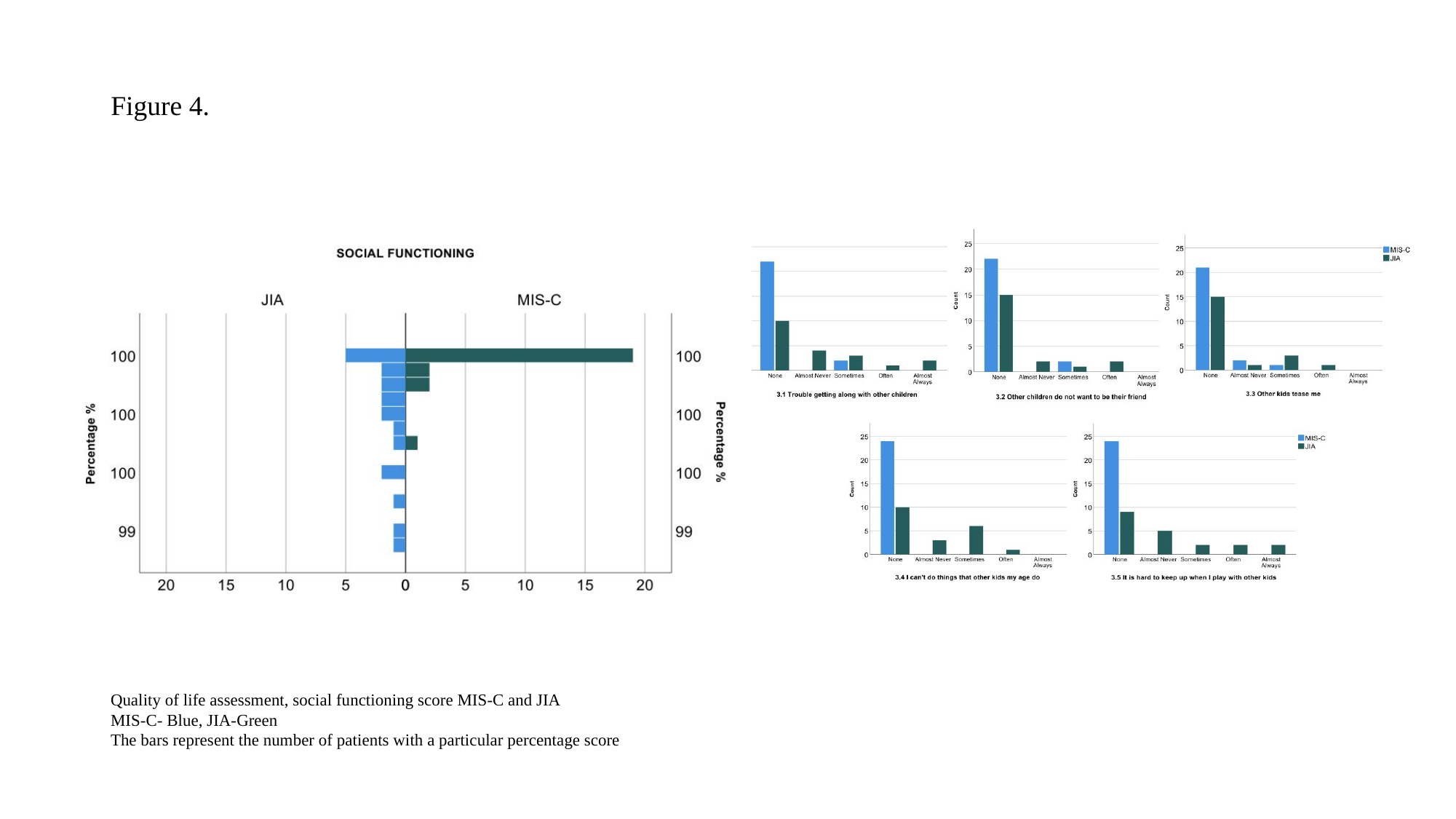

# Figure 4.
Quality of life assessment, social functioning score MIS-C and JIA
MIS-C- Blue, JIA-Green
The bars represent the number of patients with a particular percentage score

## Slide 8
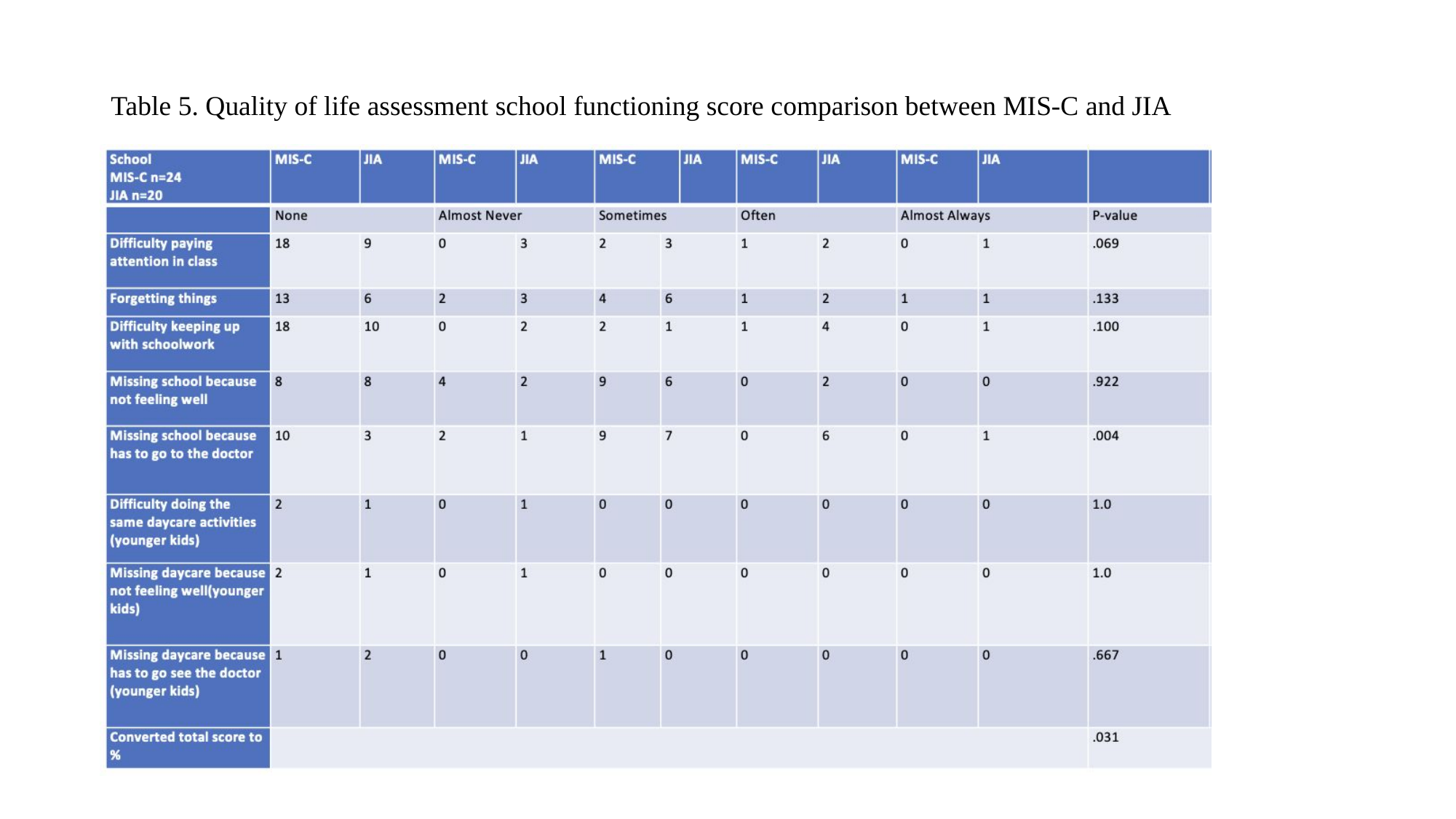

# Table 5. Quality of life assessment school functioning score comparison between MIS-C and JIA

## Slide 9
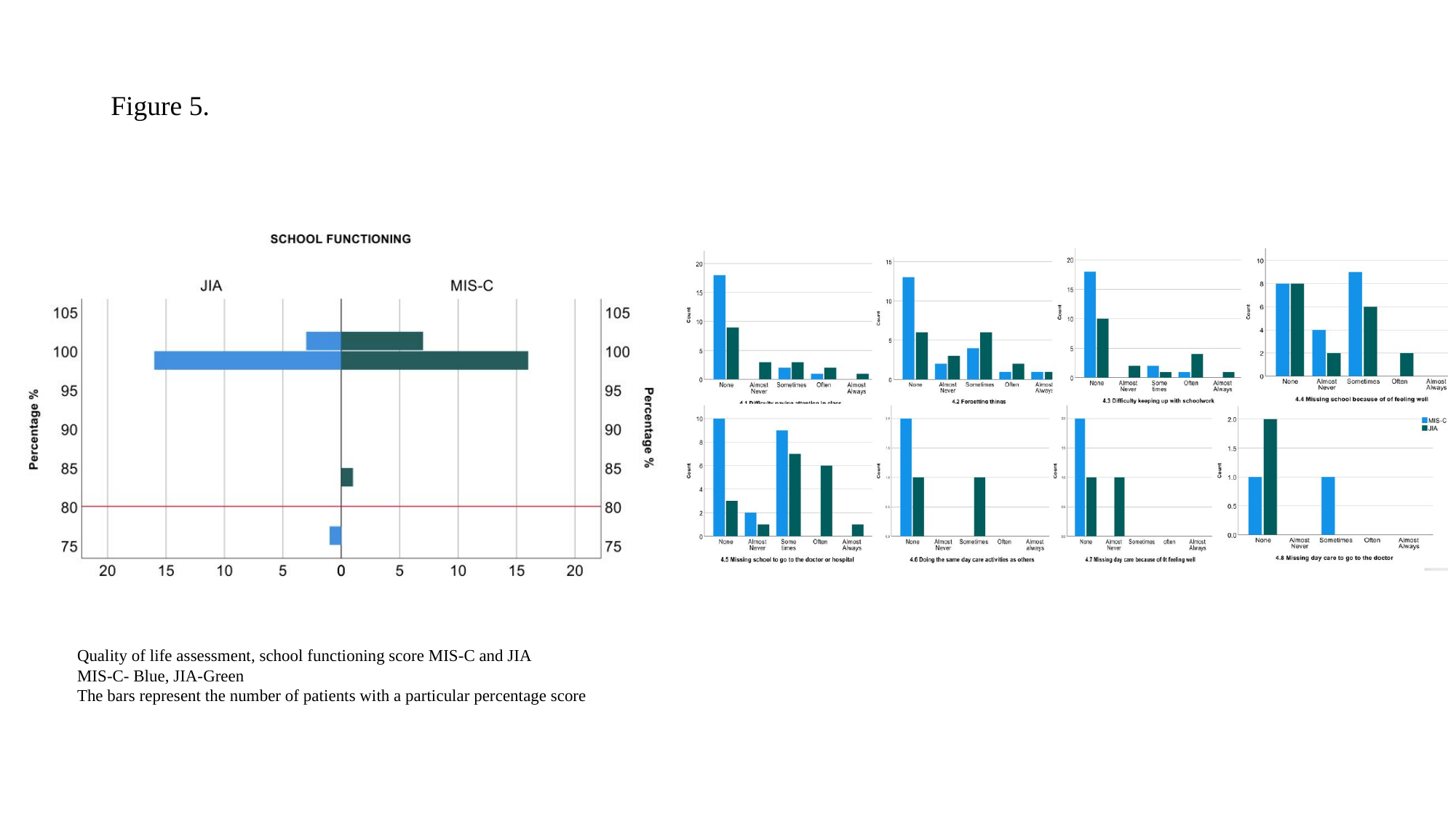

# Figure 5.
Quality of life assessment, school functioning score MIS-C and JIA
MIS-C- Blue, JIA-Green
The bars represent the number of patients with a particular percentage score

## Slide 10
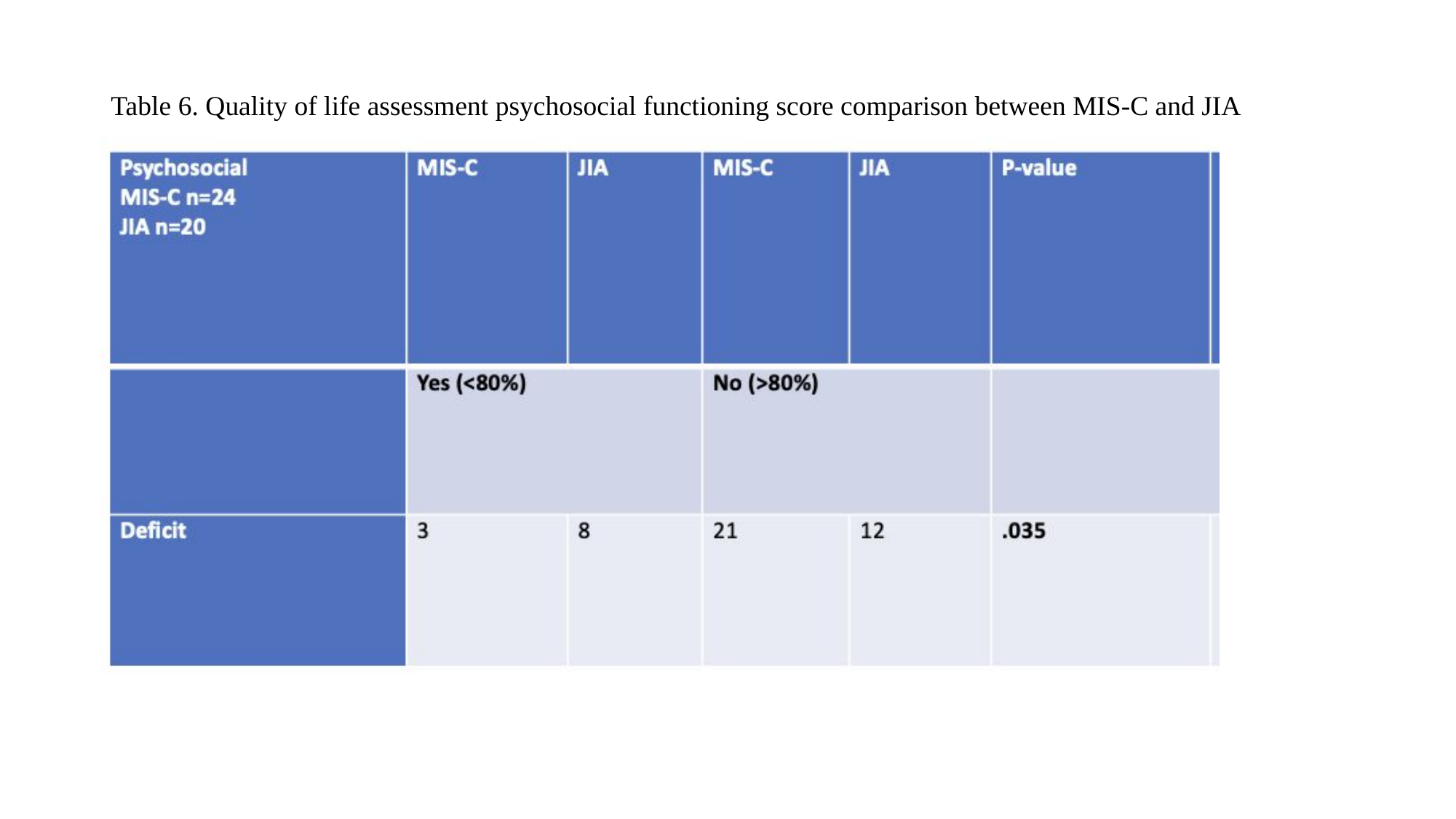

# Table 6. Quality of life assessment psychosocial functioning score comparison between MIS-C and JIA

## Slide 11
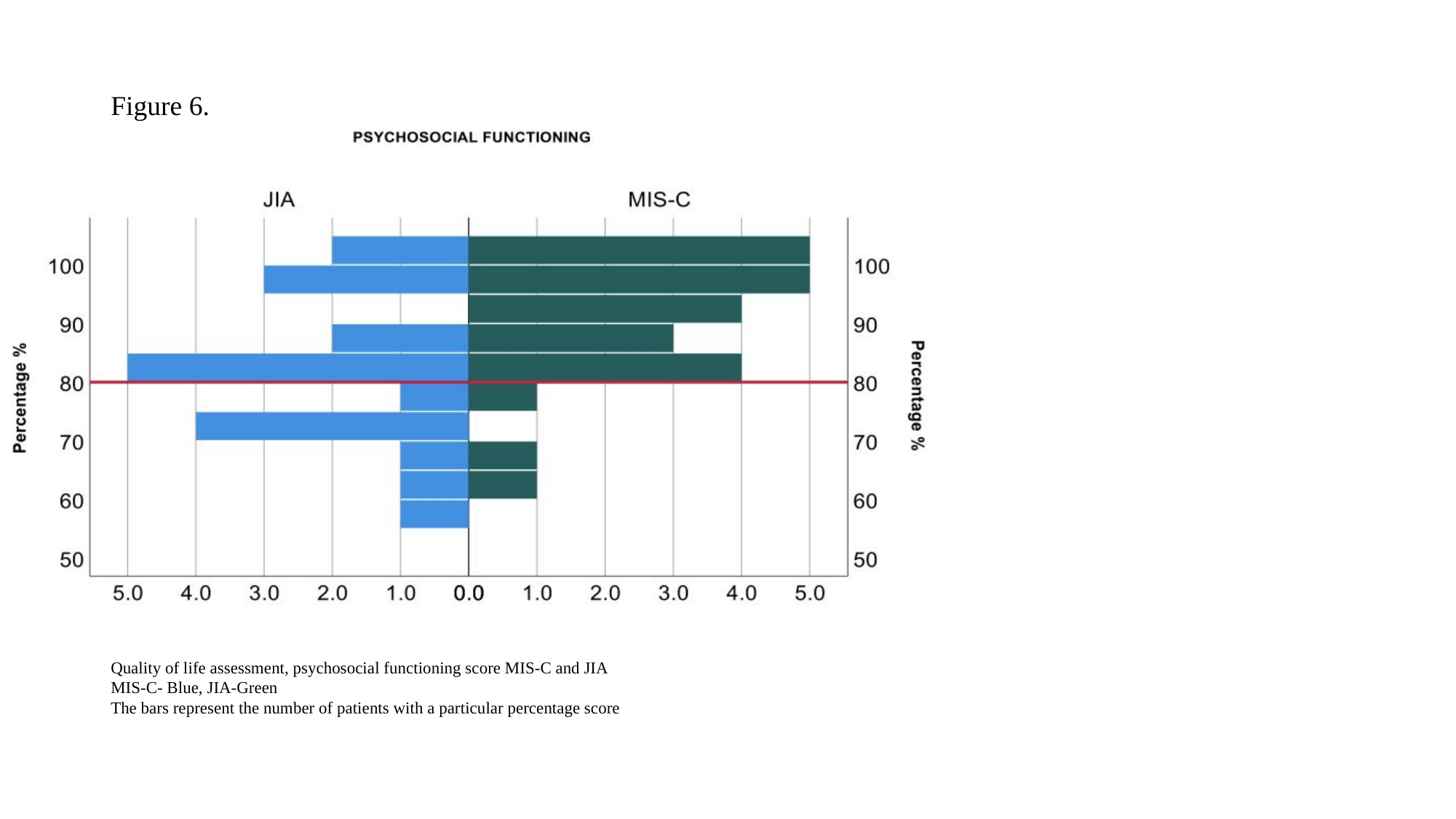

# Figure 6.
Quality of life assessment, psychosocial functioning score MIS-C and JIA
MIS-C- Blue, JIA-Green
The bars represent the number of patients with a particular percentage score
