# Supplementary material for: Medium term health and quality of life outcomes in a cohort of children with MIS-C in Cape Town, South Africa
Source: Front Pediatr. 2025 Jan 28;12:1465976. doi: 10.3389/fped.2024.1465976 (PMC11843660; doi:10.3389/fped.2024.1465976)
Supplement: Supplementary file 2 [file Datasheet1.docx]

## Appendices B1
