# Supplementary material for: Medium term health and quality of life outcomes in a cohort of children with MIS-C in Cape Town, South Africa
Source: Front Pediatr. 2025 Jan 28;12:1465976. doi: 10.3389/fped.2024.1465976 (PMC11843660; doi:10.3389/fped.2024.1465976)
Supplement: Supplementary file 3 [file Datasheet2.docx]

Case reporting form : QUALITY OF LIFE FOR MIS-C PATIENTS POST DISEASE AT THE RED CROSS WAR MEMORIAL CHILDREN’S HOSPITAL

Study number:

Date:

| DOB | Clinical complaint | BP | HR | Weight | Height | Abnormal exam findings | ECHO done |
| --- | --- | --- | --- | --- | --- | --- | --- |
|  |  |  |  |  |  |  |  |
