# Supplementary material for: Medium term health and quality of life outcomes in a cohort of children with MIS-C in Cape Town, South Africa
Source: Front Pediatr. 2025 Jan 28;12:1465976. doi: 10.3389/fped.2024.1465976 (PMC11843660; doi:10.3389/fped.2024.1465976)
Supplement: Supplementary file 4 [file Table1.docx]

Supplementary table 1

| Table 1: Essential components for the diagnosis of MIS-C | |
| --- | --- |
|  | Description |
| 1. Child | In the USA this includes adolescents up to 21 years of age. |
| 1. Fever | > 38.5^o^C |
| 1. Single OR multiorgan dysfunction | Organ dysfunction includes:  Shock, cardiac, respiratory, renal, gastrointestinal or neurological disorder  Feature may include:  Hypotension, tachycardia confusion, headache, syncope, conjunctivitis, respiratory symptoms including cough or supplemental oxygen requirement, sore throat, mucous membrane changes lymphadenopathy, neck swelling abdominal pain, diarrhoea, vomiting, rash, swollen hands and feet |
| 1. Clear evidence of inflammation | Laboratory parameters include features of an exaggerated inflammatory response and cytokine storm and include:  Raised C-Reactive protein (CRP), erythrocyte sedimentation rate (ESR), procalcitonin (PCT), fibrinogen, d-dimer, ferritin, lactic acid dehydrogenase (LDH), neutrophils, troponin T and Pro BNP.  Reduced lymphocytes and low albumin |
| 1. No clear other cause | Consider:  Bacterial sepsis, staphylococcal or streptococcal shock syndromes, infections associated with myocarditis such as enterovirus (waiting for results of these investigations should not delay seeking expert advice or management) |
| 1. SARS-CoV2 PCR testing may be positive or negative of possible antibody test should be performed | |

Reference

7. Centers for Disease Control and Prevention. Multisystem inflammatory syndrome in children (mis-c) associated with coronavirus disease 2019 (covid-19) 2020, May 14 [Available from: <https://emergency.cdc.gov/han/2020/han00432.asp>
